# Review of "*Human-Animal Medicine - Clinical Approaches to Zoonoses and Other Shared Health Risks*" by Peter M. Rabinowitz and Lisa A. Conti (eds.)

**DOI:** 10.1186/1756-3305-3-20

**Published:** 2010-03-19

**Authors:** Filipe Dantas-Torres

**Affiliations:** 1 Dipartimento di Sanità Pubblica e Zootecnia, Facoltà di Medicina Veterinaria, Università degli Studi di Bari, Strada Provinciale per Casamassima km 3, 70010 Valenzano (Bari), Italia

## Abstract

Book review of "Human-Animal Medicine, Clinical Approaches to Zoonoses, Toxicants and Other Shared Health Risks" by Peter M. Rabinowitz and Lisa A. Conti (eds.)

## Review

Although noncommunicable diseases have caused a considerable burden on public health worldwide, infectious and parasitic diseases are still responsible for an unfathomable impact on human and animal health. Moreover, with the advent of molecular biology, new infectious and parasitic diseases are continuously being discovered, and emerging zoonoses are being identified. To address this complex scenario *in toto*, a holistic and multifaceted approach is required. This brings us to the concept of "One Health Medicine", which is the order of the day in the field of public health. This concept invites public health professionals, human health and veterinary clinicians to adopt a global approach when dealing with health risks resulting from human-animal interactions. The book "*Human-Animal Medicine, Clinical Approaches to Zoonoses, Toxicants and Other Shared Health Risks*" comes to consolidate this approach, in practice.

The book is organized into 14 chapters authored by one or more of 23 contributors. The first chapter explores the convergence of human and animal medicine and the importance of environmental health. It introduces the reader to the theme, particularly to the "One Health" concept. The second chapter addresses both legal and ethical issues involved in human-animal medicine. The next chapter is intended to establish a new approach to clinical health history, providing a practical guide for human health and veterinary clinicians to identify relevant human-animal health links and potential health hazards. The fourth chapter provides sentinel signs that could serve as indicators of health risks for humans, animals, or both. The next two chapters deal with the psychosocial and therapeutic aspects of human-animal interactions as well as with the possible effects of indoor and other built environments on human and animal health. The seventh chapter broaches the allergic conditions resulting from the interactions between humans and animals. The next chapter reviews the effects of exposure to toxic hazards on humans and animals. The ninth chapter, the largest one, deals with zoonoses. Overall, this chapter is informative and the authors endeavour to provide practical recommendations for public health professionals, human health and veterinary clinicians. Most of the major zoonoses are covered, but there are some omissions, e.g. babesiosis, simian malaria, and filariasis. Perhaps, these diseases have been omitted because the book was written from a United States perspective. Indeed, this becomes more evident when the authors write "The sections in this chapter present individual descriptions of zoonotic diseases that human health and veterinary clinicians and public health professionals in the United States may encounter in their clinical work." (p. 107). Some information might also be considered out-of-date. For instance, PCR techniques for the diagnosis of leishmaniasis are said to be in development and restricted to some academic institutions, when in fact they are currently available in private veterinary laboratories, public health laboratories and leishmaniasis reference centres. The chapter tenth discusses some contemporary infectious disease scenarios, including the travel and animal contact, exotic and wildlife pets and immunocompromissed individuals. The next chapter deals with food-borne diseases affecting humans, dogs, cats and other animals whereas the chapter 12 approaches the occupational health of animal workers. The chapter 13 discusses with the role of public health agencies in the prevention and management of health risks for humans and animals. The final chapter provides practical suggestions for integrated preventive activities, at different levels (from primary to tertiary), in order to maximize human and animal health.

The book's production quality is generally good; misspellings e.g. *Amblystoma *(p. 291) and typos e.g. mtost (p. 240) are few. In some instances, species names are written without italics (pp. 198, 199). Most of the chapters contain greyscale illustrations and over a hundred colour plates are provided in the middle of the book. However, these figures are not always clear and useful, e.g. Figure 5-3 (p. 27), and sometimes repeated in different parts of the book, e.g. Figure 4-2 (p. 22) is repeated in colour as CP 9-18. References listed at the end of each chapter are generally updated. There are a few errors of fact, e.g. the brown dog tick (*Rhipicephalus sanguineus*) is stated to be the principal vector of *Anaplasma platys*, but actually there is no scientific proof on its vector role for this bacterium.

As a veterinarian with a background in public health, I was delighted to read a book providing such a holistic and multifaceted view on human-animal interactions and their possible health consequences. For public health professionals, human health and veterinary clinicians in the United States this book will be a must-read. Furthermore, I should recommend this book for all libraries of medicine, veterinary and public health schools and research centres from all over the world. This book will bring together human and animal clinicians and provide practical guidelines towards a better understanding on human-animal interactions and their potential effects on the health and wellbeing of humans and animals.

## Competing interests

The author declares that they have no competing interests.

